# Combining virtual reality and hypnosis to alleviate chronic pain in elderly with hand arthritis: protocol for a randomised phase II clinical trial

**DOI:** 10.1136/bmjopen-2025-103841

**Published:** 2025-10-05

**Authors:** Valentyn Fournier, Marie-Fania Simard, Sai Yan Yuen, Joséphine Guiné, Floriane Rousseaux, Julie Lebeau, Karim Jerbi, Philippe Richebé, Mathieu Landry, Pierre Rainville, David Ogez

**Affiliations:** 1 Maisonneuve-Rosemont Hospital Research Center, Montréal, Quebec, Canada; 2 Department of Anesthesiology and Pain Medicine, Faculty of Medicine, Université de Montréal, Montréal, Québec, Canada; 3 Division of Hematology, Oncology and Transplantation, Université de Montréal, Montréal, Quebec, Canada; 4 Department of Psychology, Université de Montréal, Montréal, Quebec, Canada; 5 Division of Rheumatology, Department of Medicine, Université de Montréal, Montréal, Quebec, Canada; 6 Department of Psychology, University of Quebec in Trois-Rivières, Trois-Rivières, Quebec, Canada; 7 Faculty of Dentistry, Université de Montréal, Montréal, Quebec, Canada; 8 CRIUGM, Montreal, Quebec, Canada

**Keywords:** Chronic Pain, Virtual Reality, Rheumatology

## Abstract

**Introduction:**

Chronic pain is a common health condition that significantly impacts the quality of life of those affected, affecting one in five people in Canada. The prevalence of this condition tends to increase with age, making it a major health issue given the ageing population. However, its management remains inadequate and requires significant mobilisation of healthcare professionals as well as the development of multiple therapeutic solutions. Among these, non-pharmacological interventions such as hypnosis and virtual reality have proven effective. Nevertheless, while the existing literature seems promising, it presents methodological limitations. Therefore, this study aims to assess the effectiveness of an intervention combining virtual reality and hypnosis in an ageing population suffering from a widespread chronic pain condition, that is, hand arthritis.

**Methods and analysis:**

This study will be a single-centre randomised clinical trial. Participants will be randomly assigned to one of two conditions: one receiving an intervention combining virtual reality and hypnosis, and the other receiving only virtual reality. The effectiveness of the intervention on current perceived pain before and after the intervention (primary outcome) will be evaluated. Secondary outcomes will include anxiety and depressive symptoms, quality of life, relaxation and fatigue. Exploratory analyses will also be conducted to contribute to the emerging literature by examining physiological variables such as heart rate variability, respiratory rate and electrodermal activity during the intervention, and their relationship with primary and secondary outcomes.

**Ethics and dissemination:**

The project was approved by the Research Ethical Committee of the Hospital Maisonneuve-Rosemont (Project no 2024-3539). Participants will be asked to provide written consent for their participation. Results from this study will be shared through peer-reviewed publications, as well as oral and poster presentations at scientific events. The protocol for this study was preregistered on Open Science Framework and raw anonymised data will be available on this platform (https://osf.io/vbh72/?view_only=1d17c5708f894faab6669d85e1fde75d).

**Trial registration number:**

NCT06833905.

**STRENGTHS AND LIMITATIONS OF THIS STUDY**

- This study considers both subjective and objective evaluations of chronic pain.
- The intervention is developed following a solid methodological framework.
- A risk for a floor effect should be considered, given the modalities of this study.
- This study will pave the way for a randomised controlled trial.

## Introduction

 Recognised as a distinct condition in the 11th edition of the International Classification of Diseases (ICD-11),^1 2^ chronic pain can be defined as an unpleasant sensory and emotional experience associated with or resembling that associated with actual or potential tissue injury.^3^ Thus, chronic pain is a subjective experience that can persist for more than 3 months and can be influenced by biological, psychological and social factors.^3 4^ Chronic pain has been reported to affect approximately one in five individuals in Canada, representing an estimated 8 million people.^5^ Considering that the proportion of individuals affected by chronic pain increases with age^6 7^ and that Canada is following a population ageing trend, it is expected that the prevalence of chronic pain and its repercussions on society will increase significantly. Projections indicate that by 2030, an additional million Canadians will be affected.^5^ Among the most common conditions leading to chronic pain in the elderly, arthritis-related conditions are prevalent.^7^

Chronic pain’s effects on psychological and physical health greatly influence a person’s quality of life. According to reports, 36% of individuals with chronic pain state that the pain is bothersome and prevents them from completing certain daily activities, thereby affecting their family and community.^8^ Because chronic pain impedes autonomy and independence, the elderly consider their pain as a top concern in their daily life.^9^ Furthermore, due to its persistent nature, chronic pain can lead to depressive and anxiety symptoms, insomnia, social isolation and may contribute to the development of mental health disorders and substance use.^5 8^

### Chronic pain management

Prolonged waiting times in public healthcare establishments for treatment of chronic pain, which can sometimes exceed 4 years,^10 11^ encourage opioids to be used as a conventional treatment because of their time-efficiency and high effectiveness for reducing pain.^12 13^ However, since the use of opioids as a treatment is, among other factors, associated with an increased risk of overdose, substance abuse and heart attacks,^14^ it is crucial to find non-pharmacological solutions to treat chronic pain and help reduce the negative outcomes of the opioid crisis in Canada.

Many non-pharmacological treatments claim to be efficient in reducing chronic pain, including meditation, clinical hypnosis and more recently, virtual reality.^15 16^ Clinical hypnosis is defined as a state of focused attention and reduced peripheral awareness resulting in an increased ability to respond to suggestions.^17^ Studies show that clinical hypnosis is an effective treatment for all four components of chronic pain, while also improving quality of life and reducing anxiodepressive symptoms and perceived pain.^18^ Virtual reality is the intentional simulation of experiences presented to the individual’s senses.^15^ This more recent intervention is also considered an effective treatment for reducing chronic pain^19^,^21^ and anxiodepressive symptoms.^22 23^ Virtual reality does so by diverting attention away from the pain experienced by individuals.^24^ Regarding the most promising type of virtual intervention, virtual nature exposure seems to be a fruitful avenue. Indeed, the effects of nature exposure are well documented, allowing for lower psychological stress and increased mood.^25^,^28^ Those effects appear to be dose-dependent, as they are maximised when people are exposed for >30 minutes/week.^29^ Given the physical limitations of patients, it appears useful to develop tailored means to bring nature to patients and virtual reality is one of them. In that sense, studies have explored the effects of virtual exposure to nature which appear to be similar to real nature exposure,^30^,^32^ demonstrating that virtual reality is a suitable solution. Considering that virtual reality and hypnosis (VRH) seem to have common mechanisms in pain management, by targeting focused and peripheral attention, combining both treatments could be an interesting approach to explore their added value in enhancing pain relief and improving patient outcomes.

### Virtual reality hypnosis

Combining VRH could increase the number of individuals who can benefit from this treatment, as this combination would address the limitations of hypnosis (ie, availability of qualified therapists, time and effort required from therapists for each patient, and patients’ ability to exert the cognitive effort needed to engage in hypnosis).^33^ The addition of virtual reality would notably reduce the cognitive effort required to generate visual stimuli associated with hypnotic suggestions, decrease the need for constant guidance from a specialised professional, eliminate the necessity of visiting a healthcare facility to receive treatment and standardise the variability in hypnotic techniques used by different practitioners.^33 34^ Therefore, a VRH intervention in pain treatment could be defined as hypnotic inductions and analgesic suggestions delivered through a personalised virtual reality software.^35^

Few studies have evaluated the effects of VRH in patients suffering from chronic pain, most of them being case studies or restricted to small samples, but results reveal positive outcomes.^36^,^38^ In a case study featuring a woman suffering from neuropathic pain, which prevented her from going outside or wearing certain clothing items, only VRH significantly improved her quality of life by reducing both the intensity and duration of her pain, compared with other ineffective treatments such as meditation, acupuncture and regular hypnosis.^36^ Other studies show that VRH not only helps reduce pain perceived by patients, but also reduces their anxiety levels.^39^ Although current studies evaluating the effectiveness of VRH as a treatment to reduce pain showcase promising results, there are only a few. They also have certain methodological limitations, including small sample sizes, no control group and are mostly focused on acute pain. Thus, to promote new non-pharmacological treatments for chronic pain, the efficacy of VRH in reducing pain perception must be evaluated by following a more rigorous methodology. Moreover, to bring comparable data on the efficacy of such an intervention on chronic pain symptoms, measurements should be comparable with those used in studies on acute pain. In that sense, considering that pain is a subjective experience of objective stimuli,^40^ physiological measures could be used to detect pain perception, as well as other indicators of quality of life-related variables.^41 42^ Most common physiological measures of pain perception are respiratory rate, heart rate, heart rate variability and electrodermal activity.^43^,^45^ Although the literature is not unanimous and mostly focused on acute pain, studies suggest that an increase in respiratory rate, heart rate and electrodermal activity, as well as a decrease in heart rate variability, is associated with higher levels of pain perception.^46^,^48^

### Objectives

The main objective of the present study is to evaluate the effectiveness of a VRH intervention in reducing pain experience in patients with chronic pain. Given the prevalence of chronic pain in the ageing population and that arthritis-related pain is among the most common conditions leading to those symptoms, this population was chosen to test the intervention. This will be achieved by comparing an experimental group, receiving the VRH treatment, to an active control group. It is expected that the subjective and physiological pain scores of individuals who received the intervention will indicate lower levels of pain than those who did not receive the intervention. It is also expected that pain scores of individuals who received the VRH intervention will show a significant reduction in pain perception between the first and last treatment session, with this effect remaining stable over time (ie, 3 weeks after the final session).

A secondary exploratory objective is to observe the impact of VRH treatment on quality of life and anxiety-depressive symptomatology. Exploratory objectives are to explore the effects of the intervention on physiological activity and on medication consumption.

## Methods and analysis

### Study design

This study is a preregistered randomised, longitudinal and monocentric project, led at the Hospital Maisonneuve-Rosemont (Montréal, Québec, Canada). This refers to a phase II randomised controlled trial according to the ORBIT (Obesity-Related Behavioral Intervention Trials) model.^49^ The goal of this type of study is to prepare the phase III study, investigating feasibility and acceptability of the study in terms of recruitment and adherence to the protocol and preliminary efficacy. According to the authors of this model, ‘pilot’ randomised trials are considered as a suitable methodology for this phase.

Recruitment of participants will be run from 1 May 2025 to 30 April 2026. Enrolment of participants will be managed by the research assistants responsible for the data collection. This study will evaluate the efficacy of an intervention combining VRH to alleviate pain in patients with hand arthritis. This study will also consider other clinical variables known to influence the experience of pain.

### Inclusion and exclusion criteria

The target population consists of individuals with inflammatory arthritis and osteoarthritis of the hands. Clubbing those two pathologies was chosen as a symptom of chronic pain, which is considered a transdiagnostic entity. Moreover, given participants are patients recruited on waiting lists in hospital, their inflammatory symptoms are controlled, leaving chronic pain at the forefront. This symptomatic approach appears reliable in this context.^50 51^ Participants will come from units of rheumatology or pain management of the Maisonneuve-Rosemont Hospital (Montréal, Québec, Canada). They receive standard medical care which can include medical treatment that will be reported among descriptive data. The inclusion criteria for both the experimental and the control group are: (a) being over 18 years old, (b) speaking and understanding French, (c) having a diagnosis of inflammatory arthritis or osteoarthritis causing invalidating chronic pain as reported by participants. The exclusion criteria include being unable to come to the hospital, having significant cognitive disorders (eg, dementia) impairing the capacity to understand and complete the questionnaires autonomously or having sensory impairments (ie, deafness, blindness) that impact the participation in the study.

### Measures

#### Primary outcome

The different dimensions of perceived pain levels will be measured using the Brief Pain Inventory–Short Form (BPI-SF).^52 53^ The BPI-SF is a self-administered nine-item questionnaire used to assess the impact of pain on patients' daily functioning. Participants are asked to rate the intensity of their worst, least, average and current pain on scales from 0 to 10, list ongoing treatments and their perceived effectiveness, and indicate the extent to which pain interferes with their usual activities. This questionnaire covers the sensory, emotional and functional aspects of the pain experience. This questionnaire has been shown to be reliable and valid in patients with osteoarthritis.^54^ The main criteria will be defined as the score of current perceived pain in the more painful hand with a higher score meaning a higher level of pain (for a psychometric justification of choosing a single-item numerical score as main outcome, see Salaffi *et al*).^55^

#### Secondary outcomes

Anxiodepressive symptoms will be measured using the Hospital Anxiety and Depression Scale (HADS).^56 57^ The HADS consists of 14 self-reported items assessing depressive symptoms (7 items) and anxiety symptoms (7 items). Participants rate their level of agreement on a 4-point Likert scale from 0 (eg, ‘not at all’) to 3 (eg, ‘completely agree’). The total score is calculated by summing responses to all item (ranging from 0 to 42), while subscores for anxiety and depression are obtained by summing responses to relevant items (ranging from 0 to 21). Higher scores indicate more severe anxiety and depressive symptoms.

Quality of life will be assessed using the 12-item SF Health Survey (SF-12).^58^ This scale evaluates eight domains of quality of life (ie, physical, social and usual activity limitations (due to physical or emotional problems), bodily pain, mental health, vitality and general health perception) categorised in two supracategories: physical component and mental component. Participants rate their agreement with various statements using various Likert scales. Higher scores mean better quality of life. After transformation of each supracategory, scores above 50 indicate a better-than-average health-related quality of life (positive outcome), while scores below 50 suggest below-average health-related quality of life (negative outcome).

Fatigue will be measured using the French short version of the Multidimensional Fatigue Inventory–10 Items.^59^ This 10-item questionnaire assesses different dimensions of fatigue (ie, physical fatigue, emotional fatigue, cognitive fatigue) on a 5-point Likert scale. Higher scores mean higher symptoms of fatigue.

At each time of measure, except the first one, a qualitative open-ended item on perceived change, asking participants whether they have noticed any change since the previous session in terms of pain, anxiety and depressive symptoms, quality of life and fatigue will be asked.

Relaxation will be assessed using a French-translated version of the Relaxation State Questionnaire (RSQ).^60^ Originally validated in German and English, this scale will undergo a translation-back translation process to ensure a reliable French version. It consists of 10 self-reported items evaluating momentary relaxation states on a Likert scale from 1 (‘does not apply to me at all’) to 7 (‘applies to me completely’). The RSQ includes four factors: muscular relaxation score, cardiovascular relaxation score, drowsiness and general relaxation score. This scale is considered as well-suited for assessing relaxation states before and after an intervention. Higher scores mean higher relaxation state.

Sociodemographic data (ie, gender, age, marital status, professional status, level of education and duration since diagnosis, diagnosis) will be collected at time 1. Gender and diagnosis will be considered for subgroup analysis, whereas age, marital status, professional status, level of education and duration since diagnosis will be considered as covariables within the analysis.

#### Exploratory outcomes

Given the intervention will be led in addition to standard medical care, information regarding antalgic, anxiolytic and antidepressant treatments will be gathered by two means: (1) by consulting medical records (ie, frequency of pharmaceutical dispensing) and (2) by asking participants what medication they consume and their weekly intake.

Physiological activity will be collected using the Biopac MP200-WS system (Biopac Systems, Goleta, California, USA), supplemented by two additional dedicated portable devices. Heart rate will be recorded using a Bionomadix RSP & ECG module (wireless transmitter and RPEC-R amplifier). Two disposable adhesive electrodes will be placed on the participants’ right and left clavicles and a third one on the chest. Heart rate variability will be derived from the data. A respiratory effort transducer (XDCR) will be used to collect respiratory rate. This belt-like device will be positioned around the ribcage at the sternum level. Finally, electrodermal activity will be recorded using the Biodynamix EDA module, which includes a plethysmograph placed on the participants’ index finger.

#### Feasibility and acceptability data

Data regarding feasibility and acceptability will be collected and reported at descriptive end. Those data will consist of recruitment rate, duration between each time of measurement and retention rate for each group. Moreover, participants will be contacted after their participation in the study to be interviewed and asked about the changes they observed in their symptoms and their point of view regarding the intervention.

### Intervention

All participants will be provided with a virtual reality headset with an integrated audio system. The Meta Quest Pro, released in 2022, will be used. A tablet connected to the headset will host the hypnosis application developed by Super Splendide (www.supersplendide.com), a company that specialises in the development of virtual reality applications. In collaboration with clinicians, researchers in the field of medical hypnosis and patient partners, Super Splendide is developing an intervention programme that combines VRH. The intervention will be delivered by one of the team members (research assistant adequately trained to communicate with participants, the intervention and the measurement tools) and supervised by VF (postdoctoral researcher in health psychology) and DO (senior researcher and clinical psychologist in pain clinics). Supervisors will be in charge of verifying the correct running of recruitments and data collection and will check those points once every 2 weeks.

For sessions 1 and 2 (ie, time 1 and time 2), participants from both the experimental and control group will not interact with the environment; they will simply be asked to watch and explore a 3D animation of a beach or a forest and relax during the session. The sounds may include the ocean, birds, wind, a crackling campfire and relaxing music. In the control group, sessions 3 and 4 (ie, time 3 and time 4) will consist of the repetition of sessions 1 and 2. In the experimental group, participants will be introduced to hypnosis techniques, the ‘magic hand’ intervention (ie, learning to apply analgesia with their hand) and to an intervention that involves manipulating their pain through an augmented reality script.

A counterbalancing procedure will be applied, respectively for sessions 1 and 2 (ie, beach or forest) and for sessions 3 and 4 (ie, magic hand or pain manipulation).

The fifth session (ie, time 5, follow-up) will correspond to a repetition of the first session (ie, time 1), the aim being to collect data in the exact same conditions to compare baseline and data collected after the intervention.

Given that cybersickness (ie, symptoms occurring when people are exposed to virtual reality) could occur, this variable will be systematically controlled for after each session in all participants with a dedicated questionnaire.^61^

### Data collection

Consent, questionnaire data and sociodemographic information will be provided by participants on a tablet using the software REDCap. The tablet will be provided by the research assistant in charge of data collection.

Participants will be included prospectively over a 6-week period. Participants will be received five times: the first four sessions (ie, time 1, time 2, time 3, time 4) occurring at a week’s interval, and a fifth session, corresponding to a follow-up session, occurring 3 weeks after the fourth session (ie, repetition of the first session).

After providing their informed consent, participants will be assigned to the previously randomly determined group (ie, the experimental group receiving the intervention or the control group) using the online tool Research Randomizer.^62^ All participants will be asked to avoid caffeinated beverages within 4 hours before the study, to avoid smoking 2 hours before the study and to avoid consuming any stimulants in general.

Participants will be received at Maisonneuve-Rosemont Hospital (Montreal, Quebec, Canada) and seated in a designated study room. This room provides a controlled, quiet environment with a comfortable seat. Before the first session, participants will complete a sociodemographic and clinical questionnaire. Participants will answer questionnaires assessing subjective variables of interest before and after each session (ie, time 1, time 2, time 3, time 4 and time 5). Physiological measurements will be taken from 10 min before the beginning of the intervention.

**Figure 1:**
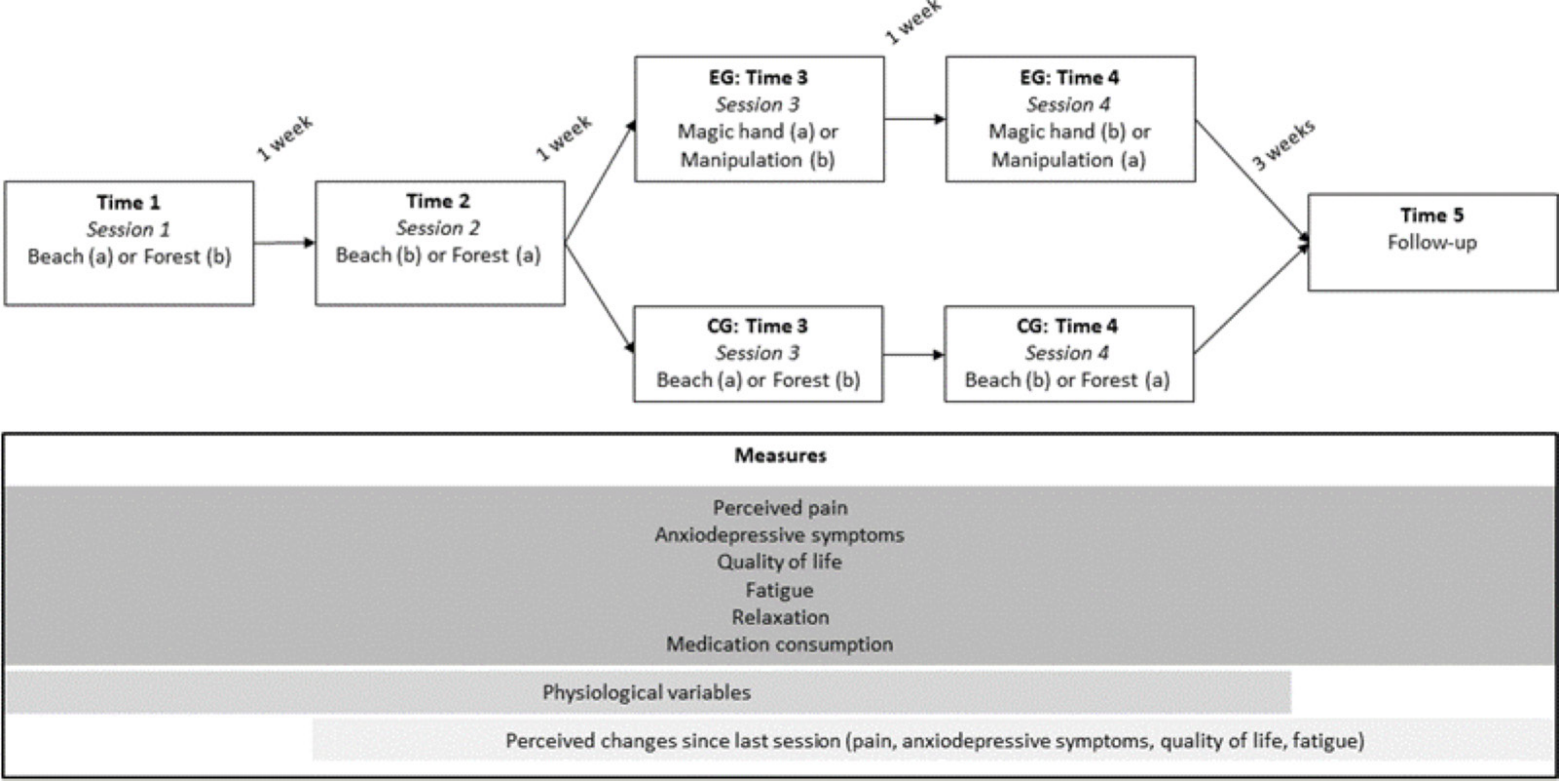
Graphical representation of the procedure.

Figure 1 displays a schematic representation of the procedure.

### Blinding and allocation concealment

Assignment to either one or the other group being fully automated and realised before the intervention by a team member who is not involved in data collection, outcome assessors will not have any influence on participants’ allocation. Allocation will be concealed until the first meeting with participants. Given the non-pharmacological nature of the study, participants and researchers directly involved in the intervention delivering and outcome assessment cannot be blinded to group allocation. Indeed, the intervention differs according to the condition and technical manipulations are needed from the assessors to display the appropriate virtual content. However, to limit remaining bias, all datasets will be coded to preserve blinding during statistical analysis. Thus, data analysts will remain blinded throughout the study and until the end of analysis.

To further reduce expectancy and performance bias, neutral information will be provided to participants, emphasising that both conditions are credible and that the study aims to evaluate which approach is more effective. Intervention materials will be presented in a standardised format, and all communication with participants will be conducted in a manner that does not suggest preferential expectations regarding one condition over the other.

### Power analysis and statistical analysis

Quantitative variables will be expressed as mean±SD and categorical variables as frequency and percentages. The impact of the intervention on outcomes of interest will be based on a univariate approach followed by a multivariate approach employing ad hoc model, depending on the structure of the data. Only variables with p<0.20 in the univariate analysis will be considered in the multivariate model. All inferential tests will be two-tailed with a significance level equal to 0.05.

An a priori power analysis was conducted for an analysis of variance (repeated measures, between factors) using the G*Power V.3.1.9.7 software considering current perceived pain as measured with the BPI-SF as main outcome. The effect size *f* was set to 0.25 (medium effect size),^63 64^ the α error risk to 0.05 and the β factor to 0.80. The resulting sample size was calculated as n=82.

As stated in the literature,^65^ an attrition risk should be considered for which an over-recruitment is recommended at time 1 to maintain the required sample size for statistical power. In that sense, a total attrition rate of 20% is expected, leading to recruitment of 98 participants at time 1. Recruitment will be stopped once this targeted sample size is reached. Missing data will be handled by proceeding to multivariable imputation.

### Feasibility

Patients from the units of rheumatology or pain management of the Maisonneuve-Rosemont Hospital will be recruited to ensure the availability of the participants. Indeed, the units are on the same floor as the laboratory where participants will undergo the intervention (for the experimental group) and where their measures will be taken, facilitating their availability for the study. From the rheumatology unit records, it is known that >3200 patients are on a waiting list for medical treatment (eg, occupational therapy) of their symptoms, showing a great availability of the target population.

A population of patients with two-hand arthritis was chosen to ensure homogeneity of the medical condition and symptoms of pain.

### Patient and public involvement

Two patients with chronic pain had a central role in the development and refinement of the application tested in this study. From the early stages, they were actively engaged in discussions among the research team to help define the application’s objectives and features. Their lived experiences brought perspective about the real needs of patients in similar conditions.

In addition, this study follows a user experience and proof-of-concept study that had led to promising results and to the refinement of the current VRH intervention. User experience data gathered direct feedback from 15 patients. Their insights were used to identify areas for improvement, leading to a redesign of key aspects of the application to enhance usability and accessibility. This collaborative approach ensured that the final version of the application was not only clinically relevant but also patient centred.

### Ethics, dissemination and preregistration

The Research Ethical Committee of the Maisonneuve-Rosemont Hospital (Project no 2024-3539) has approved this project. Any modification of the research protocol will be signalled and submitted to the Research Ethical Committee of the Maisonneuve-Rosemont Hospital.

Participants will be informed orally of the purpose of the research, the course and the duration of the study. If they declare to be interested, they will be provided with a written information electronic sheet and a written electronic consent form. They will be informed they can exercise their right to withdraw from the study at any time of completion, to access and rectify their collected data and to oppose the statistical analysis of their data.

Findings from this study are intended to be disseminated in peer-reviewed specialised professional and scientific journals. In respect to open science principles, supporting materials, raw data and analysis code will be disseminated on Open Science Framework in the ‘Phase II RCT’ folder via the following link (https://osf.io/vbh72/?view_only=1d17c5708f894faab6669d85e1fde75d). Data from participants will be kept strictly anonymised (ie, anonymisation code).

The procedure of this study has been preregistered on ClinicalTrials.gov (NCT06833905) and on Open Science Framework (https://osf.io/vbh72/?view_only=1d17c5708f894faab6669d85e1fde75d).

## Discussion

Chronic pain is a frequent and burdensome condition, with a prevalence that is expected to increase over the following decades because of the ageing population.^6 9^ Treatment of this condition remains lacunary, which appeals for the development of cost-effective solutions that do not require the constant presence of working professionals.^10^ In that sense, this study is particularly original as it explores the therapeutic potential of an emerging technology in a way that has not been extensively studied before. Its aim is to evaluate the effectiveness of a VRH intervention in reducing chronic pain among patients who are particularly vulnerable to this condition and prone to therapeutical wandering. Given the limited research on VRH and its methodological constraints (ie, small sample sizes, no control group, focused on acute pain, absence of a solid methodology of intervention development), the current study prioritises addressing these concerns and implements a longitudinal model that allows for a more comprehensive assessment of the effects of VRH on chronic pain relief. Because of its non-invasive and non-pharmacological nature, VRH holds great clinical and practical potential for chronic pain management. By being accessible in various settings, such as home-based care, hospitals and elderly care facilities, this accessible medication-free alternative treatment, that does not require continuous professional supervision, could enhance patient autonomy and facilitate broader access to pain management solutions. By advancing knowledge in this field, the findings could contribute to the development of innovative patient-centred approaches to treat chronic pain. Additionally, this research paves the way for future investigations into the long-term effects of VRH and its applicability to various types of chronic pain conditions.

One of the major limitations of this study could be the modalities of intervention itself. Indeed, literature suggests that virtual reality is effective on pain control. In that sense, all participants will benefit from an effective intervention given both experimental and control groups will have virtual reality material. In that sense, a major risk of this study is to observe only a floor effect on the different variables. Moreover, given those elements, no placebo condition is expected given no placebo VR intervention seems to be available.

The results of this phase II RCT (according to ORBIT model) are highly relevant to inform about the effectiveness of an intervention combining VRH, allowing for potential adjustments in sight of the development of a larger scale randomised controlled trial. Demonstrating the effectiveness and efficacy of the intervention will pave the way for its larger implementation in medical institutions, giving patients waiting for treatment the possibility to benefit from a non-invasive and cost-effective therapeutical solution.
