# Supplementary material for: Combining virtual reality and hypnosis to alleviate chronic pain in elderly with hand arthritis: protocol for a randomised phase II clinical trial
Source: BMJ Open. 2025 Oct 5;15(10):e103841. doi: 10.1136/bmjopen-2025-103841 (PMC12506123; doi:10.1136/bmjopen-2025-103841)
Supplement: Supplementary file 1 [file bmjopen-2025-103841.reviewer_comments.pdf]

PEER REVIEW HISTORY

BMJ Open publishes all reviews undertaken for accepted manuscripts. Reviewers are asked to complete a checklist review form and are provided with free text boxes to elaborate on their assessment. These free text comments are reproduced below.

ARTICLE DETAILS

Title (Provisional)

Combining Virtual Reality and Hypnosis to Alleviate Chronic Pain in Elderly with Hand Arthritis: Protocol for a Randomized Phase II Clinical Trial

Authors

Fournier, Valentyn; Simard, Marie-Fania; Yuen, Sai Yan; Guiné, Joséphine; Rousseaux, Floriane; Lebeau, Julie; Jerbi, Karim; Richebé, Philippe; Landry, Mathieu; Rainville, Pierre; Ogez, David

---

VERSION 1 - REVIEW

**Reviewer**	**1**
**Name**	**Varshney, Anurag**
**Affiliation**	**University of Patanjali, Allied and apllied sciences**
**Date**	**10-Jul-2025**
**COI**	**None**

Reviewer's Comments
Congratulations on bringing forward the study 'Combining Virtual Reality and Hypnosis to Alleviate Chronic Pain in Elderly with Hand Arthritis: Protocol for a Randomized Phase II Clinical Trial'. This combines two therapeutic modalities and presents a new perspective. Below are some suggestions to improve the manuscript's clarity, rigor, and alignment with journal requirements:

Major:

1. **Methodological Rigor and Validation of Intervention:** The methodology needs to be more rigorous with stronger evidence for proposed intervention mentioned in study as pilot study results are still to be out before taking up this research. The quantification of treatment modality may not be validated with mentioned outcomes. The authors should reconsider proceeding with this design until sufficient pilot data is available to justify the intervention's feasibility and measurable impact.
2. **Heterogeneous Patient Population and Outcome Measure Validity:** Clubbing both inflammatory and osteoarthritis of hand may compromise the internal validity, given the distinct pathophysiologies, symptomologies and treatment responses. Additionally, the primary outcome questionnaire used lacks demonstrated reliability in cases of inflammatory hand arthritis. The authors should either stratify these subgroups with appropriate justification or limit the study population to a more homogenous cohort. Furthermore, the outcome measures should be reconsidered or supplemented with tools that are validated across both conditions, if inclusion of both is retained.

Minor:

1. **Reference Formatting and Appropriateness:** Please ensure that the reference style strictly follows the journal's guidelines. A few references appear to be misaligned with the in-text citations or do not support the claims:
    A. The WHO reference cited in line 66, page 1, does not support the assertion that chronic pain is recognized as a "distinct disease." The cited guideline refers to chronic pain in children as a condition, not a disease. Please consider revising this claim or updating the reference.
    B. The references cited in lines 102 to 103, page 5, do not substantiate the statement regarding VR's impact on anxio-depressive symptoms and mood enhancement. Kindly verify and provide a more appropriate source.
    C. References in lines 76 to 77, page 4, are in French. While these may be valid, it is difficult to verify. If possible, please cite equivalent studies in English.
2. **SPIRIT Guidelines Compliance:** As this is a randomized trial, adherence to the SPIRIT (Standard Protocol Items: Recommendations for Interventional Trials) guidelines is essential. The following key details are missing and should be addressed:
    A. Who will deliver the interventions?
    B. Details of masking/blinding.
    C. Who will be responsible for enrolling participants?
    D. Will subgroup analyses be conducted, particularly given the inclusion of various types of arthritis (which may influence outcomes)?
    E. Who will obtain informed consent from participants?
3. **Clarification of Treatment Regimen:**
   The Methods section should clarify whether participants will receive standard medical care in addition to the Virtual Reality (VR) and hypnosis interventions, or if these interventions will be provided as stand-alone treatments.
4. **Exclusion Criteria: General Health Deterioration (Line 168, Page 8):**
   The criterion "general health deterioration" is broad. It would strengthen the protocol to specify the conditions or severity levels that constitute exclusion (e.g., specific comorbidities or functional impairments).
5. **Hand Assessment Consistency (Line 287, Page 10):**
   The inclusion of patients with arthritis in both hands should account for asymmetry in symptoms (pain, function, and deformity). Given that hand osteoarthritis often presents unilaterally, it is recommended to predefine which hand (e.g., dominant or more symptomatic) will be assessed consistently across all visits for more objective and reliable outcomes.
6. **Subjective Measures in Chronic vs. Acute Pain (Lines 339 to 340, Page 12):**
   The manuscript claims that links exist between subjective measures of acute pain and anxiodepressive symptoms or physiological data, but that such links are absent in chronic pain. Please provide a reference to substantiate this statement or consider rephrasing it if such a distinction is not well-supported in the literature.

In the end I would like to say it is commendable that the study includes an open-ended question at the end of each session to capture patient-perceived changes. This qualitative approach adds depth and patient centeredness to the outcomes assessment.

VERSION 1 - AUTHOR RESPONSE

Reviewer's Comments

Congratulations on bringing forward the study 'Combining Virtual Reality and Hypnosis to Alleviate Chronic Pain in Elderly with Hand Arthritis: Protocol for a Randomized Phase II Clinical Trial'. This combines two therapeutic modalities and presents a new perspective. Below are some suggestions to improve the manuscript's clarity, rigor, and alignment with journal requirements:

Major:

1. **Methodological Rigor and Validation of Intervention:** The methodology needs to be more rigorous with stronger evidence for proposed intervention mentioned in study as pilot study results are still to be out before taking up this research. The quantification of treatment modality may not be validated with mentioned outcomes. The authors should reconsider proceeding with this design until sufficient pilot data is available to justify the intervention's feasibility and measurable impact.

We thank the reviewer for those comments allowing us to better the manuscript.

First, the term "pilot" is related to the ORBIT framework used to develop the intervention. We acknowledge that the terms "pilot RCT" can be confusing and create misunderstandings about the goal and design of the study. In that sense, we changed for "Phase II RCT (according to ORBIT model)" and explain to what type of study it refers to (L.158-162)

> "This refers to a Phase II randomized controlled trial according to the ORBIT model [49]. The goal of this type of study is to prepare the Phase III study, investigating feasibility and acceptability of the study in terms of recruitment and adherence to the protocol and preliminary efficacy. According to the authors of the model of this model, "pilot" randomized trials are considered as a suitable methodology for this phase"

Regarding the coherence between quantification of treatment modality and outcomes, we operated to several changes within the manuscript: 1) We brought framing elements allowing us to justify to "dose" of treatment" in the Introduction (L.98-106).

> "Regarding the most promising type of virtual intervention, virtual nature exposure seems to be a fruitful avenue. Indeed, the effects of nature exposure are well documented, allowing for lower psychological stress and increase mood [25–28]. Those effects appear to be dose-dependent, as they are maximized when people are exposed for >30 minutes/week [29]. Given the physical limitations of patients, it appears useful to develop tailored means to bring nature to patients and virtual reality is one of them. In that sense, studies have explored the effects of virtual exposure to nature which appear to be similar to real nature exposure [30–32], demonstrating that virtual reality is a suitable solution"

Finally, we designed this study with patient partners and according to several pilot data (non-published or submitted) we gathered previously. This is part of a progressive and iterative development of non-pharmacological interventions as recommended by the ORBIT model. This methodology can seem disturbing given it implies a finer slicing of intervention development steps than usually. However, it allows for a precise refinement of those, responding to 1) an ethical reflection according to which interventions proposed to patients should be rigorously and methodologically sound developed and 2) an concern for efficiency. Reflections that could occur during this study or methodological flaws will be addressed during the development of the subsequent Phase III study.

2. **Heterogeneous Patient Population and Outcome Measure Validity:** Clubbing both inflammatory and osteoarthritis of hand may compromise the internal validity, given the distinct pathophysiologies, symptomologies and treatment responses. Additionally, the primary outcome questionnaire used lacks demonstrated reliability in cases of inflammatory hand arthritis. The authors should either stratify these subgroups with appropriate justification or limit the study population to a more homogenous cohort. Furthermore, the outcome measures should be reconsidered or supplemented with tools that are validated across both conditions, if inclusion of both is retained.

We thank the reviewer for this insightful comment. Indeed, we are aware that inflammatory arthritis and osteoarthritis are two distinct conditions given their respective pathophysiology. However, we have considered several points during the development of our protocol. First, we would like to adopt

a transdiagnostic vision, considering symptoms (here: chronic pain in hand(s), limitation of hand functionality) rather than considering the diagnostic entity. Besides, it should be considered that patients being recruited on the waiting list of the units, they are considered as chronic patients, stabilized in their inflammatory symptoms. Knowing that, the symptomatic approach seems suitable (Boyden et al., 2016; Das & Choy, 2023). Secondly, we are aware of the **potential** lack reliability of the primary outcome questionnaire. However, this questionnaire remains a gold standard in the literature on pain and interventional research and only the score of current perceived pain will be considered as the primary outcome (which seems to be a suitable method, according to Salaffi et al (2004)). Psychometric properties of all questionnaires will be calculated and considered along the analysis. Then, despite being secondary outcomes, we'll assess different variables (e.g., quality of life (Kosinski et al., 1999)) that could be more robust on the psychometrical level and hinder a lack of reliability that could occur. If such a case is observed, we'll consider and discuss it as a limitation in the methodology that we'll consider in the development of our future studies.

Following this comment, we plan to proceed with subgroups analysis, considering the diagnostic of patients as a covariable. If differentiated results are observed, we'll discuss it as a limitation. If no difference is observed between the group, then we'll can consider the transdiagnostic approach as adapted to this intervention (L237).

Boyden, S. D., Hossain, I. N., Wohlfahrt, A., & Lee, Y. C. (2016). Non-inflammatory causes of pain in patients with rheumatoid arthritis. Current rheumatology reports, 18(6), 30.

Das, D., & Choy, E. (2023). Non-inflammatory pain in inflammatory arthritis. Rheumatology, 62(7), 2360-2365.

Kosinski, M., Keller, S. D., Ware Jr, J. E., Hatoum, H. T., & Kong, S. X. (1999). The SF-36 Health Survey as a generic outcome measure in clinical trials of patients with osteoarthritis and rheumatoid arthritis: relative validity of scales in relation to clinical measures of arthritis severity. Medical care, 37(5), MS23-MS39.

Salaffi, F., Stancati, A., Silvestri, C. A., Ciapetti, A., & Grassi, W. (2004). Minimal clinically important changes in chronic musculoskeletal pain intensity measured on a numerical rating scale. European journal of pain, 8(4), 283-291.

Minor:

1. **Reference Formatting and Appropriateness:** Please ensure that the reference style strictly follows the journal's guidelines. A few references appear to be misaligned with the in-text citations or do not support the claims:

A. The WHO reference cited in line 66, page 1, does not support the assertion that chronic pain is recognized as a "distinct disease." The cited guideline refers to chronic pain in children as a condition, not a disease. Please consider revising this claim or updating the reference.

The reference was changed for more appropriate ones, referring to the ICD-11 rather than to the WHO (L66-68).

> "Recognized as a distinct condition in the 11th edition of the International Classification of Diseases (ICD-11) [1,2], chronic pain can be defined as an unpleasant sensory and emotional experience associated with or resembling that associated with actual or potential tissue injury [3]"

B. The references cited in lines 102 to 103, page 5, do not substantiate the statement regarding VR's impact on anxio-depressive symptoms and mood enhancement. Kindly verify and provide a more appropriate source.

The systematic review led by Wong et al. (2022) suggests the efficacy of VR for alleviate anxiodepressive symptoms. According to this comment and for clarity purpose, we reorganized this sentence and added a reference to strengthen the presented arguments. We also deleted "while improving mood" and a reference to keep the text clear (L98-99).

> "This more recent intervention is also considered an effective treatment for reducing chronic pain [19–21] and anxiodepressive symptoms [22,23]."

C. References in lines 76 to 77, page 4, are in French. While these may be valid, it is difficult to verify. If possible, please cite equivalent studies in English.

Those are federal or provincial publications. English versions were added for federal (Canada) publications but are not available for provincial (Quebec) one. We deleted the last one in consequence.

2. **SPIRIT Guidelines Compliance:** As this is a randomized trial, adherence to the SPIRIT (Standard Protocol Items: Recommendations for Interventional Trials) guidelines is essential.

The following key details are missing and should be addressed:

Indeed, following the SPIRIT guidelines will ensure transparency of this protocol. We thank the reviewer for this comment and added the information within the manuscript accordingly.

A. Who will deliver the interventions?

> "The intervention will be delivered by one of the team members (research assistant adequately trained to communication with participants, the intervention, and the measurement tools) and supervised by VF (postdoctoral researcher in health psychology) and DO (senior researcher and clinical psychologist in pain clinics). Supervisors will be in charge of verifying to correct running of recruitments and data collection and will check those point once every two weeks." (L248-252)

B. Details of masking/blinding.

> "*Blinding and allocation concealment*
>
> Assignment to either one or the other group being fully automated and realized before the intervention by a team member who is not involved in data collection, outcome assessors won't have any influence on participants' allocation. Allocation will be concealed until the first meeting with participants. Given the non-pharmacological nature of the study, participants and researchers directly involved in the intervention delivering and outcome assessment cannot be blinded to group allocation. Indeed, the intervention differs according to the condition and technical manipulations are needed from the assessors to display the appropriate virtual content. However, to limit remaining bias, all datasets will be coded to preserve blinding during statistical analysis. Thus, data analysts will remain blinded throughout the study and until the end of analysis.
>
> To further reduce expectancy and performance bias, a neutral information will be provided to participants, emphasizing that both conditions are credible and that the study aims to evaluate which approach in more effective. Intervention materials will be presented in a standardized format, and all communication with participants will be conducted in a manner that des not suggest preferential expectations regarding on condition over the other." (L290-304)

C. Who will be responsible for enrolling participants?

> "Enrollment of participants will be managed by the research assistants responsible for the data collection." (L163-164)

D. Will subgroup analyses be conducted, particularly given the inclusion of various types of arthritis (which may influence outcomes)?

"Gender and diagnosis will be considered for subgroup analysis whereas age, marital status, professional status, level of education, and duration since diagnosis will be considered as covariables within the analysis." (L221-223)

E. Who will obtain informed consent from participants?

"Consent, questionnaire data, and sociodemographic information will be provided by participants on a tablet using the software REDCap. The tablet will be provided by the research assistant in charge of data collection." (L271-273)

3. **Clarification of Treatment Regimen:**

The Methods section should clarify whether participants will receive standard medical care in addition to the Virtual Reality (VR) and hypnosis interventions, or if these interventions will be provided as stand-alone treatments.

Those elements were added (L 171-172, 225).

"They receive standard medical care which can include medical treatment that will be reported among descriptive data"

"Given the intervention will be led in addition of standard medical care (…)"

4. **Exclusion Criteria: General Health Deterioration (Line 168, Page 8):**

The criterion "general health deterioration" is broad. It would strengthen the protocol to specify the conditions or severity levels that constitute exclusion (e.g., specific comorbidities or functional impairments).

Indeed, this criterion was blurrily described. As the only functional criteria that could imply a exclusion from the recruitment, we only specified "unable to come at the hospital" (L175).

5. **Hand Assessment Consistency (Line 287, Page 10):**

The inclusion of patients with arthritis in both hands should account for asymmetry in symptoms (pain, function, and deformity). Given that hand osteoarthritis often presents unilaterally, it is recommended to predefine which hand (e.g., dominant or more symptomatic) will be assessed consistently across all visits for more objective and reliable outcomes.

This point is indeed highly relevant for this protocol and had been already considered in the methodology but poorly reported. We added this element more explicitly to the methodology. (L188-189)

"The main criteria will be defined as the score of current perceived pain in the more painful hand with a higher score meaning a higher level of pain."

6. **Subjective Measures in Chronic vs. Acute Pain (Lines 339 to 340, Page 12):**

The manuscript claims that links exist between subjective measures of acute pain and anxiodepressive symptoms or physiological data, but that such links are absent in chronic pain. Please provide a reference to substantiate this statement or consider rephrasing it if such a distinction is not well-supported in the literature.

We thank the reviewer for bringing this element to our attention. Rereading the manuscript, we concluded that this point is not essential to this part and can seem redundant we other elements in the paper. For this reason, we deleted it.

In the end I would like to say it is commendable that the study includes an open-ended question at the end of each session to capture patient-perceived changes. This qualitative approach adds depth and patient centeredness to the outcomes assessment.

Indeed, this could be valuable in our case. We considered this suggestion and added it in the protocol. (L240-242)

> "Moreover, participants will be contacted after their participation in the study to be interviewed and asked about the changes they observed in their symptoms and their point of view regarding the intervention."

Additional modifications

- The period of recruitment was modified. (L163)
